# Exhaled Air Metabolome Analysis for Pulmonary Arterial Hypertension Fingerprints Identification—The Preliminary Study

**DOI:** 10.3390/ijerph20010503

**Published:** 2022-12-28

**Authors:** Andrzej S. Swinarew, Jadwiga Gabor, Błażej Kusz, Szymon Skoczyński, Paweł Raif, Ilona Skoczylas, Kamil Jonas, Marek Grabka, Magdalena Mizia-Szubryt, Karolina Bula, Arkadiusz Stanula, Barbara Mika, Ewaryst Tkacz, Jarosław Paluch, Mariusz Gąsior, Grzegorz Kopeć, Katarzyna Mizia-Stec

**Affiliations:** 1 Faculty of Science and Technology, University of Silesia in Katowice, 41-500 Chorzów, Poland; 2 Department of Swimming and Water Rescue, Institute of Sport Science, The Jerzy Kukuczka Academy of Physical Education, 40-065 Katowice, Poland; 3 First Department of Cardiology, Faculty of Medicine in Katowice, Medical University of Silesia, 40-055 Katowice, Poland; 4 Department of Pneumonology, Faculty of Medicine in Katowice, Medical University of Silesia, 40-055 Katowice, Poland; 5 Department of Biosensors and Biomedical Signals Processing, Silesian University of Technology, 41-800 Zabrze, Poland; 6 3rd Department of Cardiology, Faculty of Medical Sciences in Zabrze, Medical University of Silesia, 41-800 Katowice, Poland; 7 Pulmonary Circulation Centre, Department of Cardiac and Vascular Diseases, Jagiellonian University Medical College, John Paul II Hospital in Krakow, 31-349 Kraków, Poland; 8 Department of ENT, Faculty of Medical Sciences in Katowice, Medical University Silesia, 40-055 Katowice, Poland

**Keywords:** pulmonary arterial hypertension, metabolome, fast screening diagnostics

## Abstract

Pulmonary arterial hypertension (PAH) is a rare disease with a serious prognosis. The aim of this study was to identify biomarkers for PAH in the breath phase and to prepare an automatic classification method to determine the changing metabolome trends and molecular mapping. A group of 37 patients (F/M: 8/29 women, mean age 60.4 ± 10.9 years, BMI 27.6 ± 6.0 kg/m^2^) with diagnosed PAH were enrolled in the study. The breath phase of all the patients was collected on a highly porous septic material using a special patented holder PL230578, OHIM 002890789-0001. The collected air was then examined with gas chromatography coupled with mass spectrometry (GC/MS). The algorithms of Spectral Clustering, KMeans, DBSCAN, and hierarchical clustering methods were used to perform the cluster analysis. The identification of the changes in the ratio of the whole spectra of biomarkers allowed us to obtain a multidimensional pathway for PAH characteristics and showed the metabolome differences in the four subgroups divided by the cluster analysis. The use of GC/MS, supported with novel porous polymeric materials, for the breath phase analysis seems to be a useful tool in selecting bio-fingerprints in patients with PAH. The four metabolome classes which were obtained constitute novel data in the PAH population.

## 1. Introduction

Pulmonary arterial hypertension (PAH) is a rare disease with a serious prognosis. Unfortunately, it is often diagnosed in the late phase as its symptoms are nonspecific [1].

Recently published studies on the molecular pathology of PAH have yielded novel data on mutations in genes associated with bone morphogenetic protein receptor type 2 (BMPR2), the role of growth factors, inflammation, hypoxia, and metabolic remodeling [2,3,4,5]. The latest research direction seems interesting, especially using novel trends in metabolome investigations [6] for patients with PAH [7,8]. The metabolic remodeling of vascular cells in PAH mainly involves energy metabolism in mitochondria [9]. It is characterized by a shift to aerobic glycolysis mediated by pyruvate dehydrogenase kinase (PDK) [10]. Several other metabolic pathways are also involved in the pathobiology of PAH, i.e., the increased metabolism of tricarboxylic acid and fatty acid oxidation [8]. They all seem to be potent PAH biomarkers when examined by the breath phase analysis, especially since, currently, there are no effective blood, or other metabolic, screening tests, which would be useful in the diagnosis of PAH.

In recent years, the analysis of exhaled air has become more and more interesting for scientists and medics. It allows for attempts to identify disease biomarkers, thanks to which it will be possible to detect diseases in the future, leading to better treatment outcomes. Various attempts to approach the issue of sampling itself, as well as its analysis, can be observed [11]. We have developed a method of collecting the respiratory phase [12].

In our previous research, we examined the serum and the breath phase of patients with PAH that was collected using a special patented holder containing a highly porous organic material, with a highly developed surface of pure carbon septic material. This novel noninvasive method seemed specific and sensitive in the range of selected bio-fingerprints [12,13].

The study aimed to identify the biomarkers for PAH in the breath phase and prepare an automatic classification methodology to determine the changing metabolome trends and molecular mapping.

The proposed method of analyzing PAH patient data consisted of two main stages.

The first stage of the analysis was to detect peaks in each patient sample (we call this sample-signal) and then to analyze the distribution of these detected peaks. This stage’s results strongly depended on the selection of the peak parameters. The parameters determine the peaks included for further analysis. This stage of the study was carried out in consultation with GCMS specialists. After selecting the peaks for each signal, the clustering of patients (detection of homogeneous clusters of patients—their signal characteristics) was started. A so-called “profile”, or a characteristic consisting of a certain number of selected features for each signal, was prepared. The vector of these characteristics was the basis for further clustering, performed using the algorithms described below. As a result of such analysis, the patients were divided into four groups. The goal of the data analysis was to propose and test such a method of analyzing data from the respiratory phase analysis (analyzed with the GCMS method), which would allow the detection of homogeneous subgroups (clusters) in the group of patients with pulmonary hypertension.

## 2. Materials and Methods

A group of 37 consecutive patients (F/M: 29/8, mean age 53.3 ± 19.8 years, BMI 27.6 ± 6.0 kg/m^2^) with diagnosed PAH and enrolled in PulmoNary HyPertension in the PoLish Population (BNP-PL)—Registry [14,15] were analyzed. PAH was established according to the ESC recommendations [1], including right heart catheterization (RHC). The following types of PAH were diagnosed: iPAH (n = 23), connective tissue disease-PAH (CTD-PAH, n = 2), congenital heart disease-PAH (CHD-PAH, n = 12), i.e., Eisenmenger syndrome (n = 5). The patients were in the following WHO functional classes (class 1—no symptoms, including no dyspnoea during exercise and rest; class 2—symptoms during activities such as climbing stairs or grocery shopping; class 3—serious limitation of the ability to perform daily activities; class 4—symptoms both during activity and during rest, including dyspnea at rest): class 1–2, class 2–19, class 3–13, class 4–3.

The mean value of NT-proBNP (diagnostic biomarker for heart failure) concentration was 1689.5 ± 3730 pg/mL (range: 62–14,585, med.: 617), the 6-min walking test (6MWT) distance was 380.5 ± 64 m.

At the moment of enrollment, the patients were on PAH-target therapy, including phosphodiesterase-5 inhibitors (PDE5i; sildenafil), endothelin receptor antagonists (ERA; bosentan, macitentan), and prostacyclin class agents (iloprost, treprostinil, epoprostenol).

Exclusion criteria involved: active inflammatory process, infectious disease, malignancy, and hematology disorders.

The study was approved by the ethics committee (Bioethical Committee, Medical University of Silesia) and was performed under the ethical standards laid down in the 1964 Declaration of Helsinki. Patients who qualified for the study gave their informed consent before their inclusion.

The respondents’ breath phase was collected on a highly porous septic material using a special patented holder PL230578, OHIM 002890789-0001. The specially designed porous organic material was prepared based on patent PL228980. The synthesis method allows for obtaining porous material with personalized sensitivity focused on breath gasses, metabolomics, and proteomics.

Before each examination, the patients rinsed their mouths with a 30% ethanol solution and then, three times with demineralized water to remove the residual ethanol.

The collected air was transported to the lab center within 2 h and then, examined with headspace analysis using gas chromatography combined with mass spectrometry (GC/MS). The analyses were performed using a Shimadzu QP 2010 Plus gas chromatograph equipped with a mass detector, autosampler AOC 5000, and a ZB 5MSI column (length 30 m, diameter 0.25 mm). Porous material with collected air was agitated at 37 °C for 30 min in a gas-tight vial. In the following step, the headspace phase was collected and injected into the GCMS system with a temperature program set from 36 °C (hold 1 min) 8 °C/min to 250 °C (hold 25 min).

The chromatographic data from patients suffering from pulmonary hypertension were processed with a signal-processing toolbox in MATLAB. In the first step, the local maxima (peaks) of the chromatogram data function were determined by their location, i.e., retention time. Only the peaks with an intensity of at least 10,000 (experimentally established) were considered. The intensity of a peak measures how much the peak stands out due to its intrinsic height and location relative to other peaks. Next, the arguments of the two neighboring local minima were determined for each found peak, and the integration range for calculating the area under each peak was established. The ratio of the area under the significant peak to its intensity was applied for further analysis. In addition, the chromatographic data were decomposed using the discrete wavelet transform (DWT) with the mother wavelet from the reverse biorthogonal wavelet family. The approximation from the first and second levels of the DWT decomposition was used to confirm the proper determination of the significant peaks in the chromatogram. The computational environment consisted of Python programming language, NumPy numerical computation library, SciPy, and Matplotlib libraries for the data analysis and visualization of the results. In addition, Jupyter Notebooks and IPython for the interactive analyses of the data and the Scikit-learn library for machine learning were used. After analyzing patient samples, creating a base of substances most often found in the breathing phase under investigation was possible. Using this database, after receiving the sample, the system will automatically give information on what substances were identified (and with what probability). The most important feature of the developed information system was that it would enable the automatic processing and analysis of data from the breathing phase samples. The system was created in the Python programming language environment (Python ecosystem).

Qualitative variables were reported as absolute numbers. Continuous variables were reported as mean ± standard deviation (SD) or median and interquartile range (IQR), as appropriate. Normality was assessed by the Shapiro–Wilk test. The statistical calculations were performed using the Statistica software version.

## 3. Results

The obtained spectral and chromatographic results present the qualitative and quantitative QA/QC sensitivity to the changes in metabolites in the patient*’*s breath.

To accomplish the first stage, the initial data preparation was performed, including, in particular: data cleaning and detection of peaks in the GCMS signals. The obtained data were used to determine the places on the retention time axis where the largest number of peaks with specific parameters (high peaks) were concentrated.

**Figure 1:**
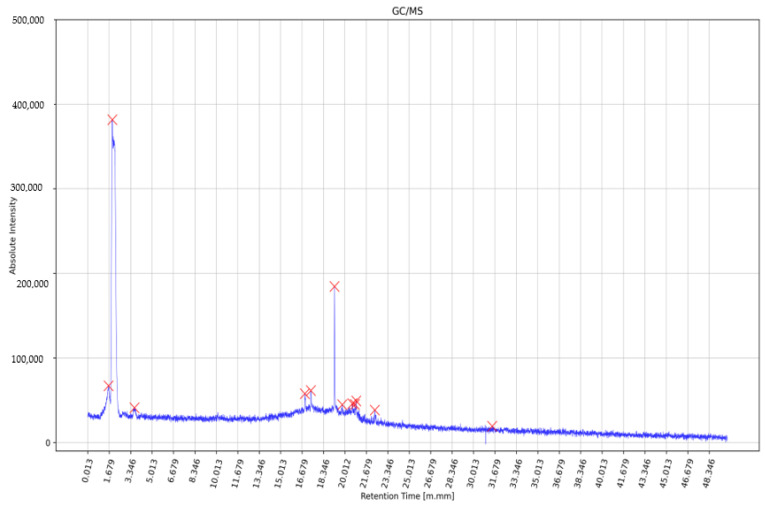
Example of the single patient data with detected peaks (depicted as red crosses).

Figure 1 shows example data for a single PAH patient. Detected peaks were depicted as red crosses on the top of each peak with specific properties; features such as intensity (height), width, prominence, and area.

**Figure 2:**
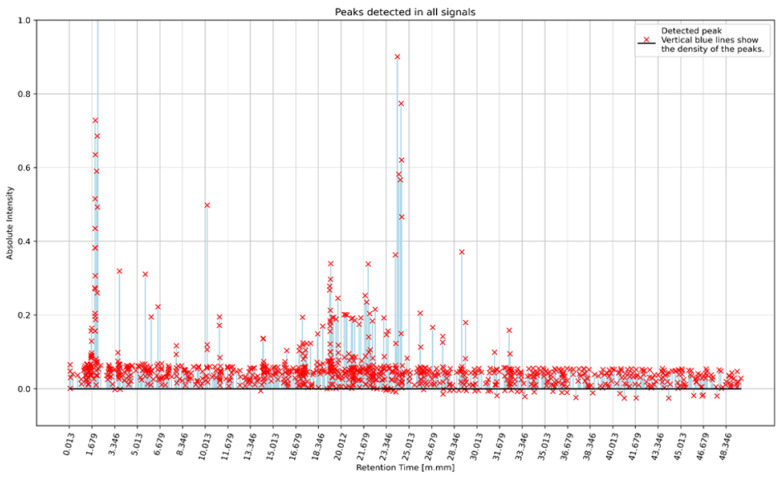
Intensity (maximum y value) and density of peaks on the x-axis in the recorded signals for the entire group of PAH patients.

**Figure 3:**
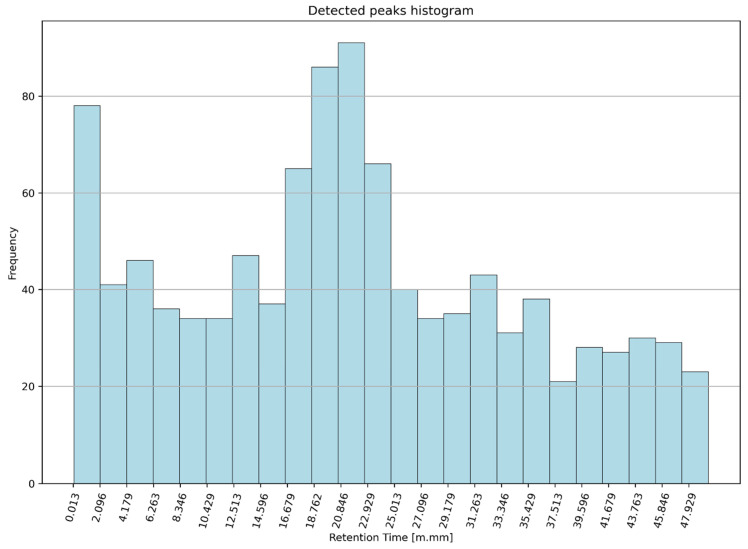
Histogram of the detected peaks for the entire group of PAH patients.

The density of the peaks in the recorded signals for the entire group of PAH patients is shown in Figure 2. The histogram of all the detected peaks for all the patients is shown in Figure 3. The data obtained in this way formed the basis for the next step: cluster analysis (clustering).

In the second stage of analysis, after the detection of the peaks in the GC-MS signals, cluster analysis was performed. The following algorithms were used: spectral clustering, k-means, DBSCAN, and agglomerative clustering (which belongs to hierarchical clustering methods). All these algorithms were implemented in the Scikit-learn library for Python programming language.

Scikit-learn implementation of a spectral analysis algorithm needs a number of classes (among other arguments, which are not relevant here). In this analysis of PAH patients, the chosen number of classes was k = 4.

**Figure 4:**
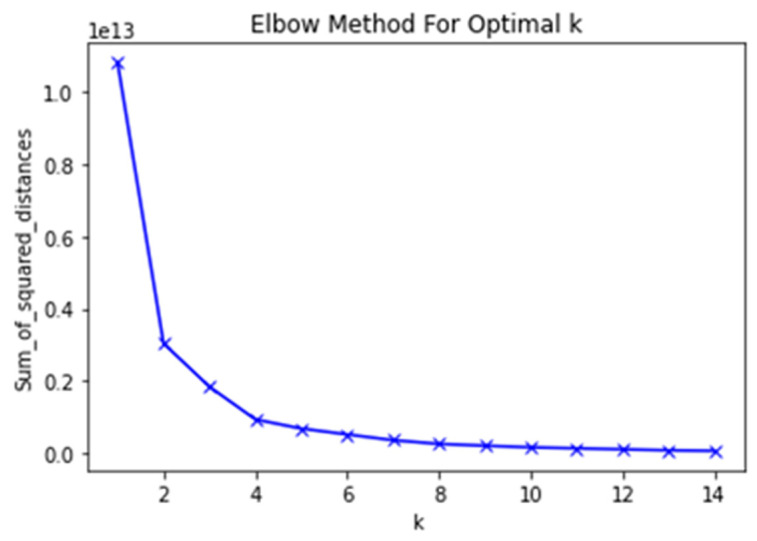
Elbow analysis plot for PAH patients’ data—determining an optimal number of clusters. The point where the curve is becoming flat—in this case, it is 4—determines the optimal number of clusters.

We used the “elbow method” to determine the optimal number of clusters in our data (Figure 4). In this method, one should choose a number of clusters so that adding another cluster does not give a much better modeling of the patient data. This was a very characteristic point, an angle in the graph; the point where the curve was becoming flat (hence the name “elbow criterion” or “elbow method”). The number of clusters was determined at this point. Adding more clusters will not bring any additional knowledge to the model.

According to this setting, in this analysis, the spectral clustering algorithm assigned patients to 4 different categories—4 clusters (Table 1). The numbers of patients in the subsequent categories were as follows: 10, 8, 11, 8. Statistical analysis of these groups of patients was performed, and the results are shown in the table (Table 2).

**Figure 5:**
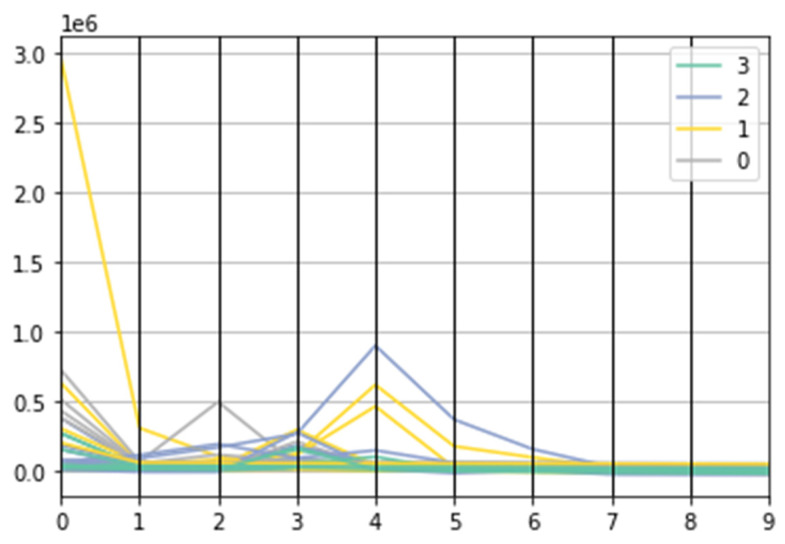
Parallel coordinates plot.

The parallel coordinates plot (Figure 5) allows for estimating which of the proposed signal features (in this case, 10) were most useful in separating a set of signals into subsets and, thus, the division of patients into groups (clusters). From the presented graph for one of the exemplary sets of parameters tested, it can be concluded that the most significant features in this situation were 0, 2, 4, 5, and then 1, 3, 6.

**Figure 6:**
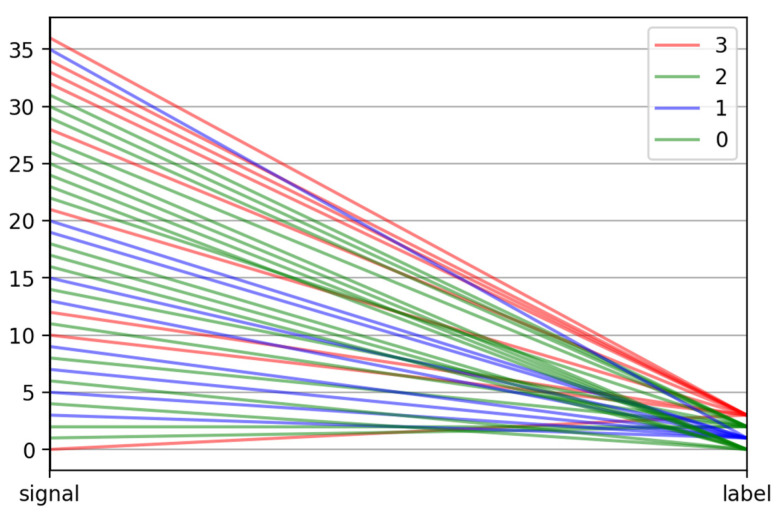
Mapping of 37 PAH patients’ data to 4 clusters marked with four colors.

Figure 6 shows an example of a clustering result visualization. The left side of the plot—the left vertical axis—shows 37 points corresponding to the patient’s data. On the right side, there are 4 data clusters. Mapping to each cluster was depicted as a line in a specific color, as shown in the figure.

The analysis of the parity composition of exhaled air in patients with PAH showed a very large variety of metabolites and their wide range. The composition of volatile compounds, from semivolatile compounds to nonvolatile compounds, absorbed through the formation of aerosols was observed. The analysis showed no specific metabolome indicative of PAH. However, it enabled the creation of clusters in which, based on the analysis using neural networks and deep machine learning, one can see the creation of multi-dimensional maps.

Table 1 presents the chemical compounds qualitatively, which are characteristic for each cluster, excluding recurrent substances compared to other clusters. A significant group of compounds was shown, characteristic and unique for each cluster, which proves that the clusters are correctly matched and selected.

The spectral clustering algorithm assigned patients to four different categories. The numbers of patients in the subsequent clusters were as follows: 10, 8, 11, 8. The clinical characteristics of the groups are presented in Table 2. There were no differences in the clinical aspects in the subsequent clusters.

## 4. Discussion

To our knowledge, this study is the first in the literature concerning the application of fast breath analysis in the metabolome characteristics of PAH. Our preliminary results produced evidence that the use of GC/MS, supported with novel porous polymeric materials, constitutes a promising method in PAH patients’ evaluation. The obtained four metabolome classes are novel, with previously unrecognized data of potent importance for PAH characteristics.

A noninvasive breath test, using polymer sampling porous brick, is one of the cheapest and easiest ways to detect cancer markers [16]. The operation’s principle is simple: the patient blows air directly into the holder, then the cartridge with the breath sample is submitted for analysis. The preliminary results showed a significant difference in the chromatograms and the mass spectra of the tested samples in the range of small molecule markers and gasses.

The occurrence of such broad and complex relationships, especially in the field of volatile and semivolatile organic compounds, and their initial analysis, indicates the reaction of metabolic gases with markers obtained directly from the lungs, further creating more complex compounds, as well as oxides and superoxides. This observation is consistent with the assumption and confirms that, in the case of the metabolome analysis, the lungs are a selective bioreactor, in which very specific, targeted reactions take place depending on the parity. This type of interpretation suggests that the extension of the research and further analysis of the obtained data, with the use of extensive neural networks, will allow for obtaining specific metabolic pathways that will be strictly dependent on the PAH phenotype of its etiology and the stage of advancement and progressive degeneration which occurred in the body.

Due to the data’s nature (multidimensionality), the spectral clustering method was a good tool for their analysis. Spectral clustering was a technique derived from graph theory [17]. This technique uses the spectrum of the similarity matrix of the data to perform dimensionality reduction before clustering in fewer dimensions. A range of a matrix was the set of its eigenvalues. In graph theory, it was used to identify possible homogenous subsets of nodes in a graph. However, it was a very flexible technique and could also be used in other data analysis tasks. The four obtained clusters also represent multidimensional maps. Identification of the mentioned cluster has been performed based on certain experimental examinations with the help of the relevant literature. Because the whole concept is strongly nonlinear, the only method left for suitable analysis refers to the application of neural networks, which tolerate the nonlinearity of the process quite well. The key point, however, is in choosing a suitable type and configuration of the network. This was based on the experience of the team members. Additionally, we have decided that the application of deep learning may fit perfectly in the attempt to find an acceptable computational solution in a relatively short time. As can be seen, the number of compounds in the identified cluster is not equal. In addition, this problem is well tolerated by the chosen tools, both neural networks and deep learning. We suspect that, based on cluster characteristics, it will be possible to accurately correlate multidimensional data not only with PAH but also with other diseases not yet identified.

The mechanism responsible for releasing a wide range of markers into the respiratory system is still unknown. However, different markers were observed in both healthy and diseased patients [18]. Probably, the markers may indicate an increased risk for developing PAH and serve as a diagnostic screening tool in the future. The presented approach may also be considered as a very first attempt at PAH identification with the help of methods belonging to widely accepted artificial intelligence.

We are aware of the limitations of our study. The most important limitation is the relatively small study group. The small sample resulted from the rare character of the disease and the number of available PAH patients in the clinical centers supporting the study. This is the preliminary study and, in our further evaluation, the number of patients will be increased in order to provide more convincing results.

All of our patients presented with PAH; however, the study group was inhomogeneous. We are aware that a PAH development mechanism is multifactorial. This can be related to genetic predisposition, congenital heart diseases, or acquired susceptibility to developing PAH in predisposing conditions [2,3,19,20]. On the other hand, both hemodynamic characteristics and target therapy are common for the whole PAH group [1]; thus, we suspected that metabolic remodeling could also be common.

In order to increase the strength of the sample, the research was conducted in a multicenter manner. The methodology of the study required analysis within two hours of the sample being taken. It constituted the next limitation in the recruitment phase of the study, i.e., the requirement for localized PAH centers.

We did not include healthy subjects as a control group in the study. In our previous observations, there were clear differences between the small and high molecular mass marker concentrations in the PAH and in the healthy groups [11,12]. Our current finding is a unique observation presented in the form of description. Taking into regard the character of the study and limitations in the metabolome cluster interpretation, the adding of the control group will not allow for a direct comparison without additional interaction.

The examined group were treated according to the ESC guidelines using PAH-specific therapies [1]. There are no data in the literature on an association of PAH-therapy and the profile of exhaled air metabolome. Unfortunately, we have no data on the exhaled air profile in untreated PAH patients and that constitutes a next limitation of the study. However, we suspect that, first of all, the clinical state of patients, i.e., WHO functional class or direct measurements in RHC may correspond with the metabolome profile.

Our study group was characterized by a female dominance that corresponds to the prevalence of PAH in the general population [1].

The lack of cardiorespiratory test results is also a limitation of our analysis.

Regardless of the limitations, the observations are unique and constitute a very promising research subject for the future.

The parity composition of exhalation includes different exhalation times and the use of breath sampling maneuvers, such as breath holding and forced exhalation, which can affect the VOC content [21,22,23]. This lack of uniformity most likely affects the reproducibility of the obtained results. However, the use of a patented device used during the test significantly reduces these inconveniences and allows for the obtaining of comparable results. The use of a neuron network will properly prepare the results with validation and will also facilitate the search in online databases for other diseases with their molecular fingerprints.

## 5. Conclusions

The use of GC/MS, supported with novel porous polymeric materials, for the breath phase analysis seems to be a useful tool in selecting bio-fingerprints in patients with PAH. The molecular level breath analysis identifies a range of biomarkers in patients with PAH. The obtained four metabolome classes, especially, constitute novel data in a PAH population; however, their interpretation is unclear and should be a step for further analyses.

## Figures and Tables

**Table 1:** Quantitative compilation of the parity composition of the exhalation phase.

**Cluster 1**
Methanone
Thymol blue
Peracetyl-[1R-(1.alpha.,4a.beta.,10a.alpha.)]-1,2,3,4,4a,9,10,10a-octahydro-1,4a-dimethyl-7-(1’-methylethyl)-1-phenanthrenem
(E)-4,5-diphenyl-2-methyl-3-pentenoic
2,3,5-Trichloro-4,6-di(p-tolyl)thiopyridine
1,2,3,4-Tetrahydroisoquinoline-N-phosphorodiamide, N,N,N’,N’-tetramethyl, 1-(2,2-dimethyl-1-hydroxypropyl)-
Diethyl Phthalate
1-Undecene, 4-methyl-
1-Bromo-4-bromomethyldecane
Sulfurous acid, dodecyl 2-propyl ester
1,2-Benzenedicarboxylic acid, dibutyl ester
Hexahydropyridine, 1-methyl-4-[4,5-dihydroxyphenyl]-
ilane, trimethyl[5-methyl-2-(1-methylethyl)phenoxy]-
4-Acetamido-N-tert-butyl-3-nitrobenzamide
3,9-Epoxypregn-16-ene-14,20-diol, 7,11,18-triacetoxy-3-methoxy-
dimethyl cis-8,8-dimethyl-5-oxobicyclo[5.1.0]oct-3-ene-1,7-dicarboxylate
Tris(2-pyridinecarboxylato-N(1),o(2))chromium(iii)
Olean-12-en-28-oic acid, 3.beta.-hydroxy-21-oxo-, methyl ester
3-Hydroxy-dl-kynurenine
tannane, trimethyl(p-neopentylphenyl)-
6-Nitro-1H-quinazoline-2,4-dione
**Cluster 2**
TRISTRIMETHYLSILYL ETHER DERIVATIVE OF 1,25-DIHYDROXYVITAMIN D2
3,5,6-TRIMETHOXY-2-METHYLCHROMONE
3-(2-Hydroxy-6-methylphenyl)-4(3H)-quinazolinone
6-Dimethyl(chloromethyl)silyloxytridecane
1-Benzenesulfonyl-1H-pyrrole
Cyclotrisiloxane, hexamethyl-
Arabinitol, pentaacetate
1,1,1,3,5,7,9,9,9-Nonamethylpentasiloxane
Cyclopentasiloxane, decamethyl-
9,10 DIDEUTERO OCTADECANAL
3-Butoxy-1,1,1,7,7,7-hexamethyl-3,5,5-tris(trimethylsiloxy)tetrasiloxane
1-Tetradecanol
QUERCETIN 7,3’,4’-TRIMETHOXY
Phthalic acid, bis(7-methyloctyl) ester
Cyclopropanetetradecanoic acid, 2-octyl-, methyl ester
ISOCHIAPIN B
Acetic acid, 17-(4-hydroxy-5-methoxy-1,5-dimethylhexyl)-4,4,10,13,14-pentamethyl-2,3,4,5,6,7,10,11,12,13,14,15,16,17-tetradec
Dotriacontane
Tetrapentacontane, 1,54-dibromo-
Phthalic acid, butyl undecyl ester
cis-1-Chloro-9-octadecene
Silicone oil
03027205002 FLAVONE 4’-OH,5-OH,7-DI-O-GLUCOSIDE
Dibutyl phthalate
2,4,6(1H,3H,5H)-Pyrimidinetrione, 5-(1-cyclohexen-1-yl)-5-ethyl-
9,9-Dimethoxybicyclo[3.3.1]nona-2,4-dione
Heptasiloxane, hexadecamethyl
Octadecanal, 2-bromo-
1,2-Bis(trimethylsilyl)benzene
1,2-Benzenedicarboxylic acid, bis(2-ethylhexyl) ester
Octasiloxane, 1,1,3,3,5,5,7,7,9,9,11,11,13,13,15,15-hexadecamethyl-
Cyclobuta[1,2:3,4]dicyclooctene, hexadecahydro-, (6a.alpha.,6b.alpha.,12a.alpha.,12b.alpha.)-
**Cluster 3**
1H,1H,2H,2H-Perfluorooctyl iodide
Benzenesulfonic acid, N’-[2-(hydroxyimino)-1-methylpropylidene]hydrazide
Me-t-BDMS of LTB4
Quinovic Acid
1,2-Thiagermolane, 2,2-dibutyl-
2-Chloro-3-(4-chloro-2-phenyloxazol-5-yl)-indol-1-carboxylic acid, 2,2,2-trichloroethyl ester
2-Dodecyl-3-methoxy-5-(2-methylpropyl) phenylester of 17-(4-Methoxyphenyl)-2,4,6,8,10,12,14,16-heptadecaoctaenoic acid
1’H-Cholest-2-eno[3,2-b]indol-6-one, 1’-(phenylmethyl)-, oxime, (5.alpha.)-
Rhodium, bis(trimethylphosphine)-chloro-.eta.2-1,2-(2,4-di-t-butyl-1,3-diphosphacyclobutadiene)-
butyl decanoate
1,3,4-Tribromo-2,5-dichlorobenzene
3-Methyl-2-(phenylselenyl)butyric acid, ethyl ester
Pentanoic acid, 4-nitro-, methyl ester
T-2 TOXIN TRIMETHYLSILYL ETHER
Podocarpan-14.beta.-ol
Benzene, 4,6-difluoro-1,2,3,5-tetrakis(phenylthio)-
**Cluster 4**
1,4-Diethylpiperazine
Chloroform
11-Methoxymacusine a
Acenaphthylene
Octasiloxane
methyl [G-(14)-C] palmitate
Butanoic acid, trimethylsilyl ester
Phthalic acid, di-(1-hexen-5-yl) ester
PROPYL 2-PHENYLSULPHONYLAMIDOACETATE
2-Acetylphenoxathin thiosemicarbazone
N-(Benzoylcarbonyl)-6-cyano-6-undecyl-2-methylpiperidine
Clomipramine
Phosphoric acid, 2,3-bis(trimethylsiloxy)propyl dimethyl ester
Stannane, tetrapropyl-
1,4-Naphthalenedione, 2,3-dichloro-
Acetamide, 2,2-dichloro-N-(2-chloro-3-pyridyl)-
6-Norlysergic acid diethylamide
5,5-Dimethyl-4-methylene-3-[2-(2-methyl-7-trifluoromethyl-1H-indol-3-yl)-ethyl]-oxazolidin-2-one
(2,4-DICHLORO-6-NITROPHENOXY)ACETIC ACID
1-Methyl-1-(3-methylbutyl)oxy-1-silacyclopentane

**Table 2:** Clinical characteristics of the subsequent clusters.

	Cluster 1	Cluster 2	Cluster 3	Cluster 4
Number	10	8	11	8
Female	7	8	7	7
Age, years	54.3 ± 12.7	54.8 ± 13.2	46.5 ± 13.9	59.9 ± 16.6
PAH-type ● iPAH ● CTD-PAH ● CHD-PAH/ES	6 0 4/3	6 1 1/1	6 1 4/1	5 0 3/0
WHO functional class: 1/2/3/4	1/5/4/0	0/4/3/1	1/5/4/1	0/5/2/1
NT-proBNP (pg/ml)	1289.1 ± 850 Range: 116–5965 Median: 501	1079.6 ± 901 Range: 95–2369 Median: 2118	2226.7 ± 4266 Range: 62–14,585 Median: 468	2061.1 ± 2281 Range: 44–5450 Median:1393
6MWT (m)	360.2 ± 77.1	338.3 ± 151	460 ± 137 (n = 10)	345.9 ± 134
Comorbidities and risk factors: ● Hypertension ● Diabetes Mellitus ● Current smoker ● Previous smoker COPD/IL	5 2 2 3 1/0	3 2 0 0 0/0	3 1 0 2 0/0	5 3 0 3 1/1
Hemodynamics: ● RAP (mmHg) ● mPAP (mmHg) ● CO (L/min) ● PVR (WU.)	5.0 ± 0.7 49.7 ± 15.6 4.67 ± 1.7 9.81 ± 6.9	11.8 ± 6.8 54.5 ± 14.5 4.89 ± 2.1 8.68 ± 4.6	5.9 ± 3.6 44.8 ± 11.7 4.72 ± 1.7 7.91 ± 5.0	5.6 ± 4.2 45.8 ± 10.1 5.30 ± 1.7 7.39 ± 3.8
PAH therapy ● PDE5i ● ERA ● Prostacyclin class agents	10 6 4 (ILO-3, TREP-1)	7 7 3 (ILO-1, EPO-2)	11 6 7 (TREP-5, EPO-2)	6 5 3 (TREP-2, EPO-1)

Data are presented as n, mean ± SD, or n (%) unless otherwise stated. iPAH—idiopathic pulmonary arterial hypertension, CTD-PAH—connective tissue disease-PAH, CHD-PAH—congenital heart disease-PAH, ES—Eisenmenger Syndrome, COPD—chronic obstructive pulmonary disease, ILD—interstitial lung disease, RAP—right atrial pressure, mPAP—mean pulmonary arterial pressure, CO—cardiac output, PVR—pulmonary vascular resistance, PDE5i—phosphodiesterase-5 inhibitors, ERA—endothelin receptor antagonists, ILO—iloprost, TREP—treprostinil, EPO—epoprostenol.

## Data Availability

The data presented in this study are available on request from the corresponding author. The data are not publicly available due to the data being about patients, they are extensive, and come from GC-MS analyses and machine learning.
